# Supplementary material for: Venomix: a simple bioinformatic pipeline for identifying and characterizing toxin gene candidates from transcriptomic data
Source: PeerJ. 2018 Jul 31;6:e5361. doi: 10.7717/peerj.5361 (PMC6074769; doi:10.7717/peerj.5361)
Supplement: Supplemental Information 3 [file peerj-06-5361-s003.gz › FinalOutput_E-20/Snaclec_botrocetin_subunit_beta_1/finaltree.pdf]

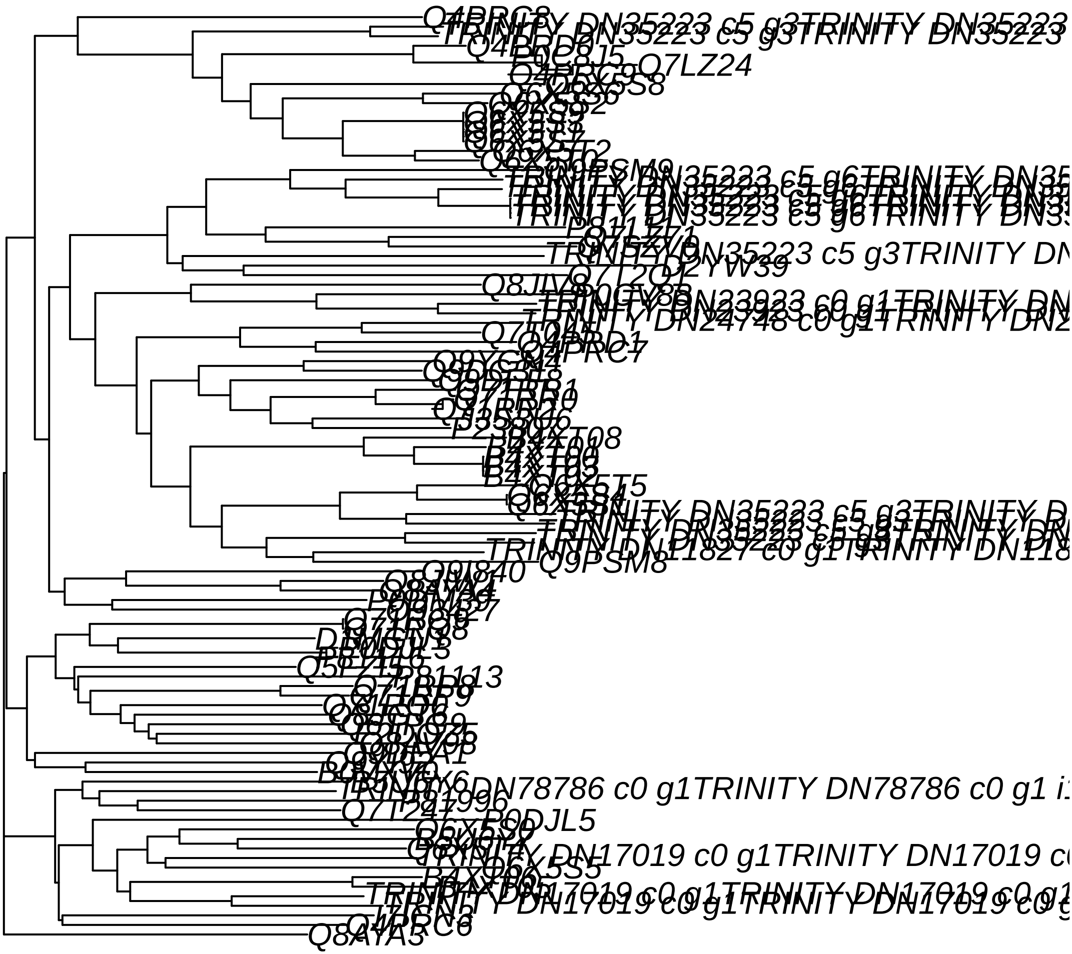
Q4PRC8
Q7LZ24
Q2YW39
Q8JIV8
B4XT08
Q9PSM8
TRINITY DN78786 c0 g1TRINITY DN78786 c0 g1 i
TRINITY DN17019 c0 g1TRINITY DN17019 c
Q8AYA3
